# Fibroblast Transplantation Results to the Degenerated Rabbit Lumbar Intervertebral Discs

**DOI:** 10.2174/1874325001711010404

**Published:** 2017-05-17

**Authors:** Ibrahim Halil Ural, Kerem Alptekin, Aysegul Ketenci, Seyhun Solakoglu, Hasan Alpak, Süleyman Özyalçın

**Affiliations:** 1 Bahcesehir University Medical Faculty, Department of Physical Medicine and Rehabilitation, Istanbul, Turkey; 2 Bahcesehir University Health Sciences Faculty, Department of Physiotherapy and Rehabilitation, Istanbul, Turkey; 3 Istanbul University, Istanbul Faculty of Medicine, Department of Physical Medicine and Rehabilitation, Istanbul, Turkey; 4 Istanbul University, Istanbul Faculty of Medicine, Department of Histology and Embryology, Istanbul, Turkey; 5 Istanbul University, Faculty of Veterinary, Department of Anatomy, Istanbul, Turkey; 6 Istanbul University, Istanbul Faculty of Medicine, Algology Department, Istanbul, Turkey

**Keywords:** Lumbar disc degeneration, Disease models, Aging, Pathology, Fibroblasts, Cells, Cultured, Animals, Rabbit

## Abstract

**Background:**

Our study is an analysis of the histological and radiological changes in degenerated lumbar intervertebral discs, after transplantation of fibroblasts in rabbits. With that study we aimed to show the viability of the fibroblasts injected to the degenerated discs, and observe their potential for further studies.

**Method:**

The apoptosis of the cell is one of the factors at the disc degeneration process. Fibroblasts may act as mesenchymal stem cells at the tissue to which they are injected and they may replace the apoptotic cells. The nucleus pulposus of the discs from eight rabbits were aspirated under scopic guidance to induce disc degeneration.

**Results:**

One month later, cultured fibroblasts, which had been taken from the skin, were injected into the disc. The viability and the potential of the injected cells for reproduction were studied histologically and radiologically. Cellular formations and organizations indicating to the histological recovery were observed at the discs to which fibroblasts were transplanted. The histological findings of the discs to which no fibroblasts were transplanted, did not show any histological recovery. Radiologically, no finding of the improvement was found in both groups. The fibroblasts injected to the degenerated discs are viable.

**Conclusion:**

The findings of improvement, observed in this study, suggest that fibroblast transplantation could be an effective method of therapy for the prevention or for the retardation of the degenerative disease of the discs.

## INTRODUCTION

Low back pain, which is a very common complaint in general population, is an important symptom because of the resulting loss of the working capacity and the increasing economic burden of the disability compensations. Complaint of a long-lasting low back pain and the pain initiating at early years are among the important causes of chronic low back pain in adults. It is reported that the incidence of the low back pain increases with increasing age. The prevalence of the low back pain begins with 7% at the age of 12 and reaches 56-67% at the age of 41 [1].

Wehling stated, “Low back pain with and without neurological symptoms remains a demanding clinical entity and has significant social and economic impact. Spinal degeneration is one factor that has been associated with low back pain but is not accessible to a cure. No surgical therapy of the spine influences the underlying pathology of degeneration in the intervertebral disc or adjacent structures of the spine. Most of the currently applied drugs mainly address pain and not degeneration. In addition, by oral administration, very little of the active drug reaches the target area. Proteinaceous mediators have great therapeutic potential and usually a high specificity of action, but are even less suited to reaching the spinal compartments.” [2].

As there is no therapeutic method to stop the disc degeneration, experimental and clinical studies have increased recently. Therefore, biochemical properties of the disc, cytokines, inflammatory answer of the disc to the stimulating agents are studied in details.

Biochemical study of the lumbar area gives us detailed information about the development and degeneration of the structures in this area and enables us to understand the alterations resulting from aging.

The intervertebral disc is basically composed of collagen, proteoglicans and water. Collagen is the basic structural protein of the body. Collagen makes up 50-70% of the dry weight of annulus fibrosus and 20-30% of the dry weight of nucleus pulposus in the disc. Collagen is not a unique protein; there may be 12 different types of collagens. There should be at least 8 types of collagen in the disc [3, 4].

Goupille stated, “approximately 80% of the collagen in the intervertebral disc is made up of fibrillar collagen Types I and II. The collagens of the nucleus pulposus and the annulus fibrosus differ to some extent, the nucleus having perhaps more in common with cartilage than with annular tissue. In the nucleus, the cells, such as the chondrocytes in hyaline cartilage, synthesize mainly Type II collagen, and only small amounts of Types VI (15-20%), IX (1-2%), and XI (3%) collagens. The intermolecular cross-links that stabilize the Type II fibrils are identical in cartilage and in the nucleus pulposus and have been shown to be of the hydroxypyridinium type. The annulus fibrosus consists mainly of Type I and II collagens, with small amounts of Type III, V (3%), VI (10%), and IX (1-2%) collagens. Type I and V collagens are synthesized by fibroblast like cells, whereas Type II collagen is usually synthesized by chondrocytes. Therefore, it is not surprising to observe nearly all Type I collagen in the outer annulus, whereas the transition zone contains virtually all Type II collagen. Very few cells are present in the intervertebral disc, approximately 1-5% of the tissue volume. The number of cells decreases rapidly across the disc from endplate to nucleus. The cells of the cartilage endplate are typical chondrocytes; those of the nucleus pulposus are similar to the chondrocytes. The cells in the outer annulus are spindle shaped and fibroblast like, whereas those in the inner transitional zone are rounded and more typically chondrocytes and resemble the cells of the nucleus pulposus.” [5].

Wehling stated, “Degenerating lumbar intervertebral discs have a destabilized or ruptured collagen and proteoglycan structure, resulting in lower water content and reduced flexibility. The lumbar disc is characterized by the presence of a low number of cells. Discal cells drive the disc metabolism and contribute to its maintenance. The nonvascularized intervertebral disc is a slowly and inefficiently regenerating tissue and at risk of becoming chronic once a pathologic situation develops.” [2].

In the recent years, intense studies are carried out in order to stop degeneration of the disc, in the areas of genetic modification, stopping the inflammatory phase, decreasing the degrading enzymes and stimulating the collagen synthesis. In this study, we tried to constitute a therapeutic method with the fibroblast injection in order to stop or slow down the degeneration of lumbar intervertebral discs.

Fibroblasts are the most easily surviving cells in the transferred environment. Their easy proliferation in the new tissue, the growth factors they secrete, the fibroblast growth factors secreted from other cells lead fibroblasts to act like stem cells and/or to stimulate the proliferation of different cell types in the environment [6]. Gruber *et al*. [7] reported that significant decrease of the discal cell number with aging and increase of the dying cells during apoptosis are the causes of failure in maintaining the optimal composition of the matrix. The same authors reported in another study that fibroblasts migrate to the area where the cells are apoptotic [8].

In the light of these information, the fibroblasts that will be transplanted to the degenerated intervertebral discs, may stimulate the regeneration of the disc with the formation of new cells, which are similar to the cells of the disc [3, 4, 6]. Furthermore, gaining the features of the chondrocytes, they may participate in the production of the collagen type II and replace the apoptotic cells, they may play a role at the internal integrity of the disc after having repaired the damaged annulus fibrosus. Therefore fibroblasts may play an effective role in the therapy of the degenerated intervertebral discs.

## MATERIALS AND METHODS

This study was planned at the Istanbul University, Istanbul Faculty of Medicine, Department of Physical Medicine and Rehabilitation. The Ethical Committee for the Laboratory Animals from the Marmara University, Faculty of Medicine, has approved the study on 22.10.2002 with the code number “54.2002.mar”.

We started with a pilot study in order to determine the method of degeneration, and to decide for the time and the frequency of follow-ups. First, front paws of the rabbits were tied upwards and they were forced to walk on the treadmill in an erect position. This method was chosen, because it is the most similar model to human disc degeneration. However, this method of degeneration could not be used in the main study due to the causes such as; unwillingness of the rabbits to walk on the mill, sliding and stepping out of the mill and getting tired quickly. The other method we tried was injecting alcohol into the disc. On this method, although we were successful in achieving degeneration, the degeneration of the first disc was seen in five days, while for the other disc it took nearly three weeks to degenerate. The method of the disc degeneration through the aspiration of the nucleus pulposus, which we used in our study, was tried on two discs in the pilot study. The degeneration of both discs was detected with the magnetic resonance imaging method (MRI) on the fifth day.

Histological examination (electron and light microscopy) and the radiological examination (magnetic resonance imaging) were used as the evaluation methods of the study. These criteria were chosen as the criteria of joint evaluation recommended by Kurunlahti *et al*. [9]. The assessments were made at the baseline, at the end of the first month and six weeks after cell transplantation. The health parameters of animals were; loss of body weight, decrease of food and water intake, weakness, infection, paralysis and behavioral changes.

Eight male, New Zealand albino rabbits, at the age of one, with weight varying between 2.5 and 4 kg that were obtained from the laboratories of the Faculty of Veterinary were used in the study.

**Fig. (1):**
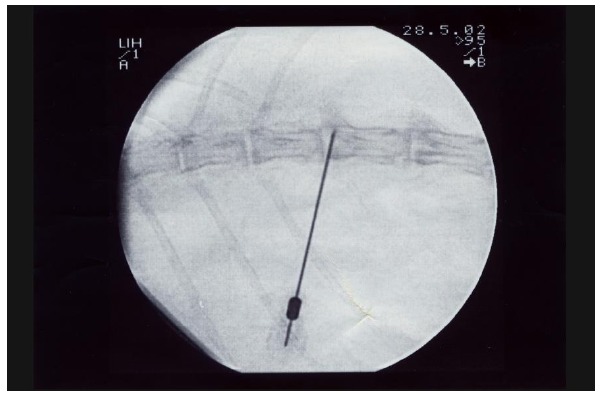
Mechanic degeneration induced via scopic control.

The first lumbar MRI’s of the rabbits were taken under general anesthesia in the Radiology Department in order to see their basic lumbar vertebral state. Three days after this examination, the nucleus pulposus of the discs T_12_-L_1_ and L_1_-L_2_ were aspired with lumbar vertebral biopsy needle (Spinocan^®^ No: 22) under scopic control, and general anesthesia, to induce mechanic degeneration, in the Skill Development Laboratory of the Anesthesia and Reanimation Department (Fig. **1**). At the same session, a skin piece of 1x1 cm, which had been shaved and cleaned with 70% alcohol before, was taken out with its subcutis by the method of punch biopsy from the dorsal area, and the wound was closed surgically. The skin biopsies were transferred in sterile Petry dish to the laboratory of the Histology and Embryology Department, in order to proliferate fibroblasts in tissue culture. The dermis and the subcutis, whose epidermis had been separated with scalpel, were chopped approximately into 0.5mm^3^ pieces and after that, they were shaken for 1 hour in buffer solution containing 1mg/ml collagenase B (Roche^®^ diagnostics Collagenase B 1 088 807) and 0.1mg/ml DNAase (Roche^®^ diagnostics DNAase I 104 159) at 37ºC. After the surface liquid was removed with centrifuge, the material was washed with the medium. Then, the medium was incubated for three weeks in 25cm^2^ flasks containing 10% FCS (Fetal Calf Serum) in Dulbecco’s Modified Essential Medium (DMEM)+HAMS F-12 medium at 37ºC temperature and at the environment containing 5% CO_2_+95% Air composition. The confluent cells were gathered with the tripsination method.

Animals were followed-up for one month, underwent the second MRI under general anesthesia in order to determine the condition of the intervertebral discs and to determine the level of the degeneration. Three days after this examination, the fibroblasts produced from the skin were injected into the degenerated T_12_-L_1_ discs, calibrated as 8,000,000cells/ml, in 0.5ml medium under scopic control and general anesthesia. L_1_-L_2_ discs were left degenerated.

Antibiotic prophylaxis was performed 30 minutes before, 24 hours and 48 hours after every procedure in three doses of 250mg ceftriaxon, intramuscular. Before and after every procedure, the weight of the animals was measured and their neurological examination was made. For general anesthesia, 35mg/kg ketamin was administered as the anesthetic agent. The level of the anesthesia was controlled by the answers given to tail pinching and extremity drawing.

After 6 weeks of follow-up, the third MRI was performed to examine the state of the discs, the level of the degeneration and to see whether there is a recovery in the degeneration.

Three days after this procedure, euthanasia was performed to the rabbits under general anesthesia, with an injection of 40mg/kg Pentobarbital-Na into the ear vein. After it was determined that there is no more beat of the apex and that the pupillary light reflex is negative, columna vertebralis between the T_12_-L_4_ was excised surgically and soft tissues, lateral and spinous processes were removed. Afterwards the T_12_-L_1_ disc (degenerated and fibroblast injected) and the L_1_-L_2_ disc (degenerated but not fibroblast injected) and the L_2_-L_3_ (not degenerated) control disc were excised completely with the scalpel and they were fixed in 2.5% glutaraldehyde for the electron and light microscopic examinations. Under the light microscopy, 10 areas were studied in every histologic specimen and the number of the cells was counted.

SPSS 7.5 was used for statistical analyses. We applied “Kruskal-Wallis” test.

## RESULTS

The findings were examined in six aspects:

1. Cell type
2. Number of cells
3. Morphology of cells
4. Organization of collagen fibers
5. The electron microscopic findings
6. Radiological findings

**Fig. (2):**
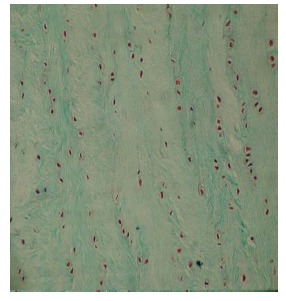
Electron microscopic findings of normal control disc.

**Fig. (3):**
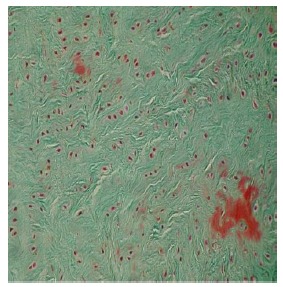
Electron microscopic findings of degenerated and fibroblast injected disc.

**Fig. (4):**
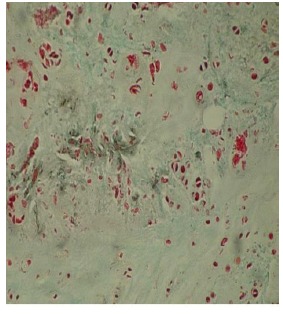
Electron microscopic findings of degenerated only disc.

The results were obtained through the examination of the normal control discs (Fig. **2**), of the degenerated and the fibroblast injected discs (Fig. **3**) and of the degenerated-only discs (Fig. **4**).

The findings of the undegenerated intervertebral discs (L_2_-L_3_ discs, Group 1) were used as the control results for the degenerated discs. The number of the cells with the fibroblast morphology in this group was 27.62±6.63. The number of the chondrocytes was determined as 26.35±10.27 and the number of the mitotic chondrocytes as 0.48±0.08.

In the injected area of the degenerated disc (T_12_-L_1_ discs, Group 2), the number of cells with fibroblast morphology, which were produced in the culture medium, was 55.37±11.00. The number of chondrocytes was 83.25±13.22; and the number of mitotic chondrocytes was 16.75±1.50. Chondrocytes forming the annulus fibrosus in the periphery were located as well organized lines between collagen fibers. The collagen fiber bundles displayed the morphology of normal annulus fibrosus.

The number of cells displaying fibroblast morphology in the injection area of the discs, which were degenerated but not injected with the cultured fibroblasts (L_1_-L_2_ discs), was 25.15±3.10. The number of chondrocytes in this group was determined as 70.35±3.25, whereas the number of mitotic chondroblasts was determined as 16.25±1.50. The studied cells in this group displayed a disorganized and partially degenerated appearance. These were scattered, and at some parts, broken bundles of collagen. The general picture of the degenerated discs consisted of disorganized bundles of collagen fibers, which formed the annulus fibrosus and most of the cells did not show the normal morphology of chondrocytes. We observed some cells with picnotic nuclei, and the cytoplasmic integrity of the cells had disappeared.

At the light microscopic examination of the second and third groups, we observed intense mitotic activity compared to healthy discs. The statistical tests revealed that the fibroblast number of the second group was significantly higher (p<0.001) than the fibroblast numbers of the third and the control groups. The number of chondrocytes in the second group was only significantly more than the control group (p<0.001). The numbers of fibroblasts and chondrocytes were not significantly different compared to the control group. The number of mitotic chondroblasts in the second and the third groups was significantly different compared to the control group (p<0.001) (Table **1**).

**Fig. (5):**
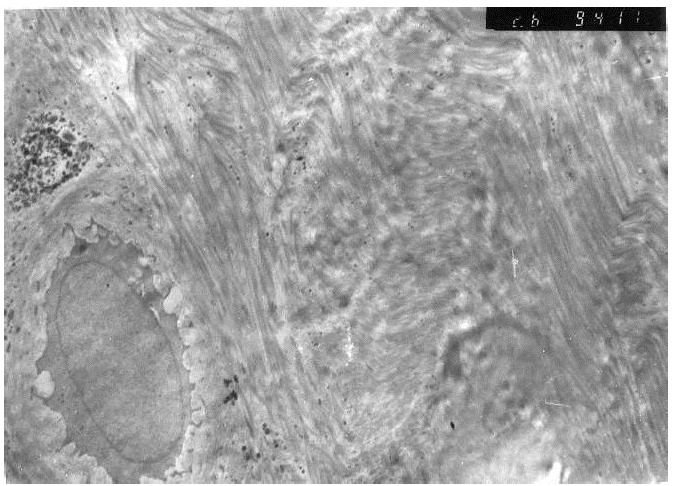
Chondroblasts of the second group investigated under the electron microscopy.

Nuclear euchromasis was observed in the chondroblasts of the second group investigated under the electron microscopy (Fig. **5**). This finding indicated that the cell was at the replication, in other words, at the proliferation phase. The collagen fibers were arranged in angles with each other, in accordance with the typical fibrous cartilaginous formation.

**Fig. (6):**
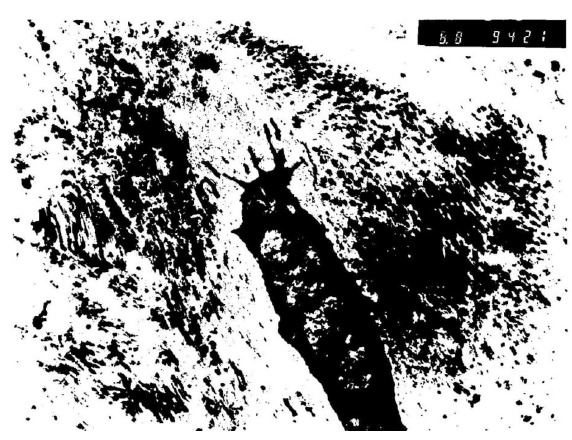
Nuclei of the studied cells of third group displayed a heterochromatic appearance under the electron microscopy.

In the third group, the nuclei of the studied cells displayed a heterochromatic appearance (Fig. **6**). This means that the cells were in an inactive phase of the synthesis. Cytoplasmic walls of the most fibroblasts of this group could not be seen and the cytoplasm contained degenerated cell remnants. Collagen fibers were scattered around the degenerated cartilage cells without forming organized bundles.

**Fig. (7):**
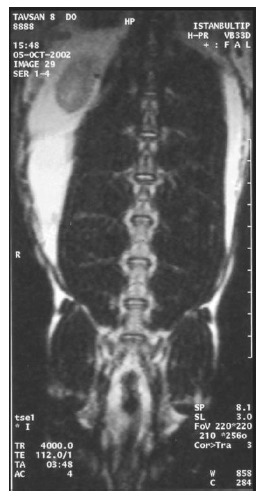
Healthy discs on MRI.

**Fig. (8):**
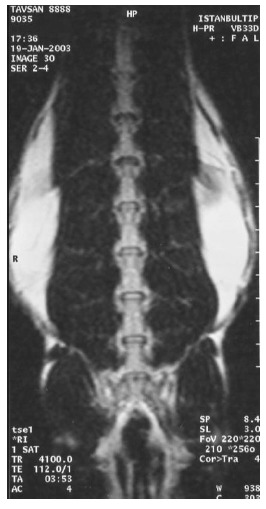
Degenerated discs on MRI.

**Fig. (9):**
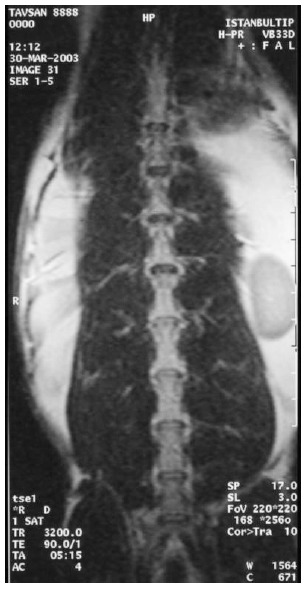
Discs after fibroblast injection on MRI.

At the first MRI's, we observed that intervertebral discs and vertebral bodies of all the rabbits were healthy (Fig. **7**). After the degeneration of intervertebral discs, the degenerated T_12_-L_1_ and L_1_-L_2_ discs of all the rabbits displayed a collapsed appearance, at the second MRI (Fig. **8**). At the third MRI examination, which was made after the fibroblast injection, no radiological recovery of degenerated intervertebral discs could be observed (Fig. **9**).

## DISCUSSION

Most of the studies concerning the disc degeneration are made on the whole spine or they are conducted at postmortem. Therefore, neither consecutive alterations, nor changes at a special area can be observed. A few degeneration models have been developed for investigative disc degeneration studies. One of these models consists of the injection of hyaluronidase or chemopapain into the disc. Lindblom produced experimental disc degeneration in rats by hanging them from their tails for a couple of weeks so that they take a ‘’U” form. Another commonly used method is to incise the annulus of the rabbit disc surgically from the ventral side. Formerly, only morphology and histology were examined by this method. Lipson and Muir also investigated the alterations of proteoglycans after an injury and the recovery periods, by this method. During this study, they observed that the annulus recovered by forming a scar tissue in three or four weeks after the surgery and that except the incised ventral area, other areas of the disc started to disorganize and degeneration increased in time. The investigators reported that the disc degeneration was completed in six months [10]. We chose the method of mechanical degeneration of the disc by needle aspiration. This method had been used at the study of Nishimura and Mochida [11]. By this method, disc degeneration took place quickly by both mechanical disintegration of the annulus fibrosus fibers and aspiration of the nucleus pulposus.

Disc degeneration is seen at the tissue level in terms of progressive loss of proteoglycans in the matrix; this component contributes to the “shock absorber” biological function of the disc. Annular fissures and tears may also reflect changes in matrix remodeling or collagen composition. Proteoglycans, collagens, and matrix remodeling are the results of activities of the disc cell population(s). The decrease in the number of cells in the disc, which occurs by aging and degeneration, also has an impact on disc matrix maintenance. Thus, the volume and activity of the cellular population within the disc are both extremely important [12].

In the light of the information, the studies about cell transplantations gained speed in the recent years. Gruber *et al*. showed that the ‘’sand rats” were a good model for autologous disc cell injections [12]. In another study, investigators showed that the disc cells could be induced to produce different types of collagen and extracellular matrix products with the help of different carriers and they reported that it could be possible to prevent degeneration if the cells could be transplanted into the discs. These studies emphasized the investigation of cell lifetime and finding out whether these cells would be able to fulfill their duty of mechanical support [13]. In our study, some of the fibroblasts injected into the disc also transformed into cells resembling the chondrocytes and they enhanced the production of the chondroblasts. These cells survived for six weeks. As this was a preliminary study, we planned a longer duration of follow-up, biomechanical evaluation, cell injection into different discs and the evaluation of collagen type for our next study.

Okuma, Nishimura and Mochida showed with their study that the developing degeneration after disc herniation could be retarded by autologous transplantation of the nucleus pulposus [14]. But autologous transplantation of the nucleus pulposus into the degenerated disc is complicated, because the extraction of sufficient nucleus pulposus from the healthy intervertebral disc may cause degeneration of the donor disc. In the study of Nomura **et al*.,* it is showed that transplantation of the nucleus pulposus cells with the extracellular matrix gives better results than isolated transplantation of the nucleus pulposus cells [15]. Therefore, it was observed that the extracellular matrix produced by the cells of the nucleus pulposus plays an important role in retarding the degeneration of discs. They also observed that the synthesis of collagen type II in the inner parts of the annulus fibrosus increases and the production of cells similar to chondrocytes increases as well. The investigators observed the retardation of disc degeneration during the follow-up period of 16 weeks. They reported that the increase of collagen type II indicated the degeneration and that the existence of cells similar to chondrocytes was a sign of regeneration, so that these two results are in discordance. Osti *et al.* declared that the collagen type II is an important indicator of regeneration [16, 17]. As the study of Nomura *et al*. lasted for 16 weeks, they do not support the results of the study, which was conducted by Osti *et al*. for 4-6 weeks. We observed in our study that the recovery period of the degenerated disc already started 6 weeks after the fibroblast transplantation. We concluded that the cells resembling chondrocytes, which were also observed by Nomura *et al*., are those that initiated the recovery and that they have similarities as the cells we observed. If proliferation and organization of these cells can be kept on, it is possible to prevent the degeneration of intervertebral discs.

We did not undertake the determination of the collagen types. The chondrocytes producing lines, which form an organization histological similar to the control group, lead us consider that this as the start of the annulus fibrosus repair. According to the statistical analysis made at the end of our study, it seems that fibroblasts acting like stem cells in discs, where they had been injected, together with growth factors in the milieu may play an important role in the number of fibroblasts and in the organization of cells in the discs. The significant results of the chondrocytes in the second group compared to the control disc showed us that the transplanted fibroblasts also stimulated the proliferation and the organization of chondrocytes. In spite of the increase in mitotic proliferation of the discs that were not injected with fibroblasts (Group 3), the chondrocytes did not show the normal morphology of chondrocytes, which indicated to the important role of fibroblasts in the repair of tissues, in terms of proliferation and cell organization.

Compared to the control group, an intense and statistically significant increase in the mitotic activity was observed in both of the degenerated intervertebral discs. But the irregular organization of the cells in the third group and the regular organization of the cells in the second group were explained with proliferation characteristics of the fibroblasts in this milieu and with their capacity to stimulate the proliferation of the regional cells [6].

Nishimura *et al*. experimentally produced disc herniation in a study conducted on 112 rats [11]. After dividing the rats into three groups, they aspired the nucleus pulposus from a healthy disc and injected it into the herniated disc in the first group. The second group of rats was injected the nucleus pulposus aspired two weeks before and the third group of rats was injected silicon gel or sodium-hyaluronate instead of the nucleus pulposus. The histological examination at the end of the study revealed the retardation of degeneration in the first group of discs and although the degeneration of discs in the second group also was delayed, this delay was less than the first group. However, the investigators reported that the degeneration phase in both groups continued although it had been retarded. In the third group, no signs of recovery or delay of degeneration could be observed. The results of this study are similar to our findings. We consider that the cells similar to chondrocytes that were observed by the investigators during the histological examination are the same chondroblasts, chondrocyte groups and lines we observed. The investigators proposed that the growth factors are secreted into the environment following the transplantation of the nucleus pulposus and that these growth factors are playing an important role at retardation of degeneration. Probably, these factors play a significant role in the recovery findings of our results. In the study of Nishimura *et al*., the extraction of cells from the healthy disc and their injection into the degenerated disc means traumatization of that segment as well. But in our study, we could obtain the fibroblasts without traumatization of the healthy discs.

In an *in vivo* study, Thompson *et al*., demonstrated that some growth factors stimulated the proteoglycan synthesis in the nucleus pulposus and they concluded that the disc would possibly be able to regenerate with the help of these growth factors [18]. Although growth factors promise some therapeutic hope, even with the newest technologies it is not possible yet to ensure homogenous dispersion of cell proteins, especially in chronically ill patients. An alternative solution to this problem may possibly be to induce cells with genetic modifications in order to enable them secrete their growth factors autonomously.

As derived from this hypothesis and with the help of genetic modification, Nishida *et al*., enabled nucleus pulposus cells that they produced in the culture medium, to secrete growth factor [19]. Conclusively, they observed that a reparation mechanism developed around these cells. The investigators considered that such a method could possibly give rise to an immune reaction. But at the end of the study, they did not observe any inflammatory reaction. The investigators concluded that the unique anatomical and physiological structure of the disc is the principal cause for nondevelopment of any inflammatory reaction. Also in our study, we did not observe any inflammatory cell infiltrate in the microscopic examination; which made us consider that no inflammatory reaction developed.

Some of the investigations aiming to stop the disc degeneration are the genetic studies. Wehling *et al*. proposed the loss of the proteoglycans as one of the most important steps of disc degeneration and they indicated to the importance of cytokines, especially the interleukin-1 and the tumor necrosis factor [2]. They suggested that intervertebral disc degeneration could be stopped or cured if sufficient cells can be placed into the target tissue, with the injection of a suspension, containing a mixture of cells, which are obtained from the vertebral end plate or intervertebral disc and which included analgesic or protective genes or genes with both features. For this reason, Wehling *et al*. obtained the vertebral end plate cells from the coccyx of the cattle and they produced the proliferation of the interleukin-1 receptor antagonistic protein in these cells. The investigators concluded that genetic methods could be used for the therapy of the intervertebral disc degeneration, with the help of the exogenous therapeutic gene transfer method into the intervertebral end plate cells. Although the therapeutic objectives of our study were similar to those of Wehling *et al*., the methods and the results are completely different. As gene therapies are expensive and not easily accessible, this study is considered most probably to stay experimental and the practical use for patients is still very difficult. The superiority of our study lays in its results obtained from live tissues.

Besides preclinical studies with rabbit or rat models, there are also few studies targeting human disc tissue. A recent study investigated human umbilical cord mesenchymal stem cell transplantation in two chronic discogenic low back pain cases. After careful disinfection and centrifugation processes cord tissue was cultured and prepared for intradiscal transplantation. Both patients suffered from back pain and discography revealed that back pain originated from degenerated discs. Either disc herniation or inflammatory diseases accompanied disc degeneration. After the procedure patients were followed for two years. In follow-up, a statistically significant decrease in Visual Analog Scale and Oswestry Disability Index was observed and no adverse events occurred [20].

Another study experienced NuQu allogenic juvenile chondrocytes delivered percutaneously for lumbar spondylosis. As NuQu uses juvenile chondrocytes, the authors suggested these cells as immune-privileged. The cells are frozen before the procedure, thawed before the procedure and combined with fibrin sealent carrier. 15 patients were treated with percutaneous delivery of NuQu juvenile chondrocytes. 13 patients were followed at 6 months with Oswestry Disability Index, Numerical Rating Scale, SF-36 and MRI. All scores improved significantly from baseline. Out of 13 ten patients indicated improvements in MRI. Convalescence of high intensity zones and disc contour and height was observed. No disc infections and serious adverse events were noted. The findings sustained in 8 patients for a follow up of 12 months [21].

## CONCLUSION

In conclusion, the fibroblasts taken from the rabbit skin and injected into the disc, continued to live and proliferate in this environment and they stimulated the proliferation of the chondrocytes. This advancement, resulting in the reparation of the traumatized annulus fibrosus, may be helpful to reorganization of internal integrity of the disc. The long-term follow-up of the injected cells and the observation of the regeneration phase of the disc should be among the objectives of the future studies. Therefore, other studies with longer follow-up periods, with larger samples including biomechanical evaluation, injection into different discs and the determination of collagen type, should be planned.

## Figures and Tables

**Table 1:** Comparison of number of chondrocytes and mitotic chondroblasts regarding all groups.

		**Number of fibroblasts**	**Number of chondrocytes**	**Number of mitotic chondroblasts**
Group 1	L_2-_L_3_ groups	27.62±6.63	26.35±10.27	0.48±0.08
Group 2	T_12_-L_1_ groups	55.37±11.00	83.25±13.22	16.75±1.50
Group 3	L_1-_L_2_ groups	25.15±3.10	70.35±3.25	16.25±1.50
	p	<0.001	<0.001	<0.001
		(G2-G1 and G2-G3)	(G2-G1)	(G2-G1 and G3-G1)
		**Number of fibroblasts**	**Number of chondrocytes**	**Number of mitotic chondroblasts**
Group 1	L_2-_L_3_ groups	27.62±6.63	26.35±10.27	0.48±0.08
Group 2	T_12_-L_1_ groups	55.37±11.00	83.25±13.22	16.75±1.50
Group 3	L_1-_L_2_ groups	25.15±3.10	70.35±3.25	16.25±1.50
	p	<0.001	<0.001	<0.001
		(G2-G1 and G2-G3)	(G2-G1)	(G2-G1 and G3-G1)
		**Number of fibroblasts**	**Number of chondrocytes**	**Number of mitotic chondroblasts**
**Group 1**	**L_2-_L_3_ groups**	**27.62±6.63**	**26.35±10.27**	**0.48±0.08**
**Group 2**	**T_12_-L_1_ groups**	**55.37±11.00**	**83.25±13.22**	**16.75±1.50**
**Group 3**	**L_1-_L_2_ groups**	**25.15±3.10**	**70.35±3.25**	**16.25±1.50**
	**p**	**<0.001**	**<0.001**	**<0.001**
		**(G2-G1 and G2-G3)**	**(G2-G1)**	**(G2-G1 and G3-G1)**
